# Proteomic analysis of the cerebrospinal fluid of patients with restless legs syndrome/Willis-Ekbom disease

**DOI:** 10.1186/2045-8118-10-20

**Published:** 2013-06-07

**Authors:** Stephanie M Patton, Yong Won Cho, Thomas W Clardy, Richard P Allen, Christopher J Earley, James R Connor

**Affiliations:** 1 Department of Neurosurgery, Penn State University, Milton S. Hershey Medical Center, 500 University Drive, Hershey, Pennsylvania PA 17033, USA; 2 DongSan Medical Center, Keimyung University, Taegu, Korea; 3 Bayview Medical Center, Johns Hopkins School of Medicine, Baltimore, MD, USA

**Keywords:** Sleep disorders, Restless legs syndrome/Willis-Ekbom disease, Nitric oxide, Hypoxia inducible factor, Pain

## Abstract

**Background:**

Restless Legs Syndrome/Willis-Ekbom Disease (RLS/WED) is a sensorimotor disorder that causes patients to experience overwhelming and distressing sensations in the legs compelling the patient to move their legs to provide relief. The purpose of this study was to determine if biomarkers in the cerebrospinal fluid can distinguish RLS/WED patients from neurological controls.

**Methods:**

We obtained CSF samples by lumbar puncture from 5 early-onset RLS/WED patients and 5 controls. We performed 2-dimensional difference in-gel electrophoresis (2D-DIGE). Proteins that were significantly altered were identified by Student’s t-test. Protein spots that were differentially expressed (*p* ≤ 0.05, Av. Ratio ≥ 2.0) between RLS/WED and control CSF samples were identified using MALDI-TOF-MS. Statistical analyses of the validation immunoblot assays were performed using Student’s t-test.

**Results:**

In this discovery study we identified 6 candidate CSF protein markers for early-onset RLS/WED. Four proteins (Cystatin C, Lipocalin-type Prostaglandin D2 Synthase, Vitamin D binding Protein, and β-Hemoglobin) were increased and 2 proteins (Apolipoprotein A1 and α-1-acid Glycoprotein) were decreased in RLS/WED patients.

**Conclusions:**

Our results reveal a protein profile in the RLS/WED CSF that is consistent with clinical findings of disruptive sleep, cardiovascular dysfunction and painful symptoms. Moreover, protein profiles are consistent with neuropathological findings of activation of hypoxia inducible factor (HIF) pathways and alterations in dopaminergic systems. These data indicate the CSF of RLS/WED patients may provide information relevant to biological basis for RLS/WED, treatment strategies and potential new treatment targets.

## Background

Restless Legs Syndrome/Willis-Ekbom Disease (RLS/WED) is a sensorimotor disorder that affects between 5-10% of the population [1]. Patients who suffer with RLS/WED experience an overwhelming and distressing sensation that forces them to move their legs [2]. Those with moderate to severe symptoms report significant disability, chronically reduced sleep times, 20% reduction in their work productivity, diminished quality of life, and increased depression and anxiety [3]. RLS/WED has also been identified as a risk factor for cardiovascular disease [4-7]. RLS/WED prevalence increases with age and it affects women twice as frequently as men.

Iron dysregulation in the pathogenesis of RLS/WED is supported by a substantial volume of research including several studies reporting decreased ferritin in cerebrospinal fluid (CSF) and decreased brain iron particularly in the substantia nigra using MRI, ultrasound imaging and brain autopsy analysis [8]. A number of studies to date support the concept of diminished brain iron in RLS/WED, thus providing the basis for the hypothesis that RLS/WED occurs as a result of low brain iron content [8-10]. We still do not understand, however, the full consequences that low brain iron may have on neural systems in patients with RLS/WED. We performed proteomic analyses in order identify neural systems and pathways that are altered in those suffering with RLS/WED and possibly identify novel avenues for potential therapeutic intervention.

CSF was utilized for this biomarker study because it is most likely to reflect changes in CNS metabolic status due to its proximity to the brain. An advantage of CSF as the biological fluid for proteomic analyses over blood is that the CSF is sequestered behind both the blood–brain and brain-CSF barriers. This isolation permits the identification of biomarkers that are specific to CNS disease processes. Hence, our goal for this study was to identify CSF biomarkers for RLS/WED using two-dimensional difference-in-gel electrophoresis (2D-DIGE) together with tandem Mass Spectrometric (MS) analysis. To further validate the identified biomarkers, we used immunoblot analyses to quantify differences in protein levels between early-onset RLS/WED and age- and gender-matched control subjects. In this study, we identify six proteins whose levels are altered in early-onset RLS/WED subjects.

## Methods

### Patient demographics and CSF collection

The study was approved by DongSan Medical Center Institutional Review Board in Taegu, South Korea where subjects were recruited and CSF was collected. The two-dimensional Difference-In-Gel Electrophoresis analyses were performed at Penn State Hershey College of Medicine. The diagnosis of RLS/WED was made by a board certified Korean neurologist with expertise in RLS/WED diagnosis (YWC). The diagnosis was based on diagnostic standards set by the NIH workshop on RLS/WED [2] and was made during a face-to-face interview utilizing questionnaire from the validated Korean-language version [11] of the validated John Hopkins Telephone diagnostic questionnaire [12] in order to exclude RLS mimics and peripheral neuropathy. All RLS/WED subjects in this study were diagnosed with primary RLS (i.e., no subjects had RLS due to other causes such as peripheral neuropathy, neurodegenerative disorders, multiple sclerosis, drug use, and/or chronic renal failure). All of the RLS/WED subjects were required to have the onset of symptoms prior to the age of 35. All of the RLS/WED were asked about a family history of RLS but having a positive family history of RLS was not required for inclusion and no family history of RLS/WED was reported by any study participants (Table 1). Control subjects were matched for age and gender and the demographics are shown in Table 2. The controls lacked any major medical, psychiatric or neurological disorders and had to answer “no” on the first two questions of the RLS/WED Diagnostic Questionnaire [11,12]. Both RLS/WED and control subject participants were screened for obstructive sleep apnea through sleep questionnaires. In addition, all subjects (both RLS/WED and controls) had to meet the following criteria: 1) no dopaminergic, anti-convulsant, or opioid medication use within the previous 6 weeks; 2) no sedating anti-histamine or short-acting sedatives (GABA-active) medication use within the previous 4 weeks; 3) no use of psychiatric medication including anti-depressants or dopaminergic medication; although hypnotics were not excluded as standard hypnotics have little effect on iron or dopamine levels; 4) no evidence of abnormal liver function (as measured by elevated serum glutamic oxaloacetic transaminase (SGOT), elevated serum glutamic-pyruvic transaminase (SGPT), elevated bilirubin, or elevated alkaline phosphatase values); 5) no evidence of an abnormal complete blood count (CBC); 6) no evidence of an abnormal blood urea nitrogen (BUN) or creatinine value; 6) no evidence of low hemoglobin (<11); 7) no history of blood transfusion or donation within the last 6 months; 7) BMI <35; and 8) no reported use of iron or other metal supplements within the 6 weeks prior to the study.

**Table 1:** **RLS**/**WED patient demographics**

**Subject number**	**Onset age (years)**	**Family history**	**Neuropathy**	**Sleep apnea or snoring**
RLS/WED 1	27	negative	negative	negative
RLS/WED 2	30	negative	negative	negative
RLS/WED 3	34	negative	negative	negative
RLS/WED 4	20	negative	negative	negative
RLS/WED 5	35	negative	negative	negative

**Table 2:** **Ages of RLS**/**WED and control subjects at time of study**

**Subject number**	**RLS/WED (age in years)**	**Control (age in years)**	
1	49	54	
2	68	63	
3	74	70	
4	48	51	
5	51	56	
	Mean ± SD	Mean ± SD	p-value
	58.00 ± 12.10	58.80 ± 7.66	0.904

After written informed consent was obtained from the research subjects, approximately 10 mL of CSF was obtained via lumbar puncture with a 25-gauge needle and placed immediately on ice. CSF specimens were spun at low speed in a refrigerated centrifuge to remove any cellular contamination, aliquoted, and stored at −80°C within 1 h. All samples were collected between 9:30 AM and 11:00 AM. The CSF samples were precipitated with trichloroacetic acid/acetone following storage at− 80°C and the protein pellets were resuspended in 10 μL of DIGE labeling buffer (7 M urea, 2 M thiourea, 4% 3-[(3-cholamidopropyl)-dimethylammonio]-1-propanesulfonate (CHAPS) and 30 mM Tris/HCl, pH 8.5). Protein levels were quantified using the 2D Quant-Kit (GE Healthcare, Uppsala, Sweden).

### Materials

Isoelectric pH gradient (IPG) strips, urea, 3-[(3-cholamidopropyl)-dimethylammonio]-1-propanesulfonate (CHAPS), dithiothreitol (DTT), Pharmalyte pH 3–11, acrylamide, ammonium persulfate, TEMED, glycine, methylene bisacrylamide, TRIS, thiourea, and 2-D Quant Kit (PlusOne or Ettan™ sample preparation kits and reagents) were obtained from GE Healthcare (Piscataway, NJ, USA). ReadyPrep 2D-Clean-up Kit was obtained from Bio-Rad (Hercules, CA, USA). Iodoacetamide and mineral oil were obtained from Sigma (St. Louis, MO). SDS was obtained from USB (Cleveland, OH, USA). Methanol and glacial acetic acid were purchased from Fisher Scientific (Fair Lawn, NJ, USA).

### Two-dimensional difference-in-gel electrophoresis (2-D DIGE) labeling

Each of the dry CyDyes (GE Healthcare) was reconstituted and 50 μg of total protein was labeled with each diluted CyDye solution as described in the manufacturer’s protocol.

### 2-D PAGE analysis

CSF containing 150 μg of labeled protein was added to an IPG strip. Strips were rehydrated for 20 h followed by isoelectric focusing for 27 h. The following gradient program was used – step 1: 0.01 h, 150 V, 2 mA, 5 W; step 2: 2.00 h, 150 V, 2 mA, 5 W; step 3: 6.00 h, 500 V, 2 mA, 5 W; step 4: 5.00 h, 3500 V, 2 mA, 5 W; step 5: 9.20 h, 3500 V, 2 mA, 5 W; step 6: 5.00 h, 50 V, 2 mA, 5 W. SDS-PAGE was carried out in 12.5% of acrylamide. Following the focusing step, the proteins on the strip were reduced and alkylated with gentle shaking in two subsequent steps for 30 min each in 10 ml equilibration buffer (6 M Urea, 75 mM Tris–HCl pH 8.8, 29.3% glycerol, 2% SDS, 0.002% bromophenol blue) containing 100 mg DTT and 250 mg iodoacetamide in the second step. Strips were removed from equilibration solution, blotted, and placed on a 1-mm thick 25.5 × 20.5 cm 12.5% acrylamide non-gradient SDS gel. The strips were sealed with 1% agarose, 125 mM Tris–HCl at pH=6.8, 1% SDS. Proteins were separated on the gels using 600 V, 400 mA, 100 W for 4 h at 10°C. Analytical gels were imaged immediately after separation, while the preparative gels were fixed with 7.5% acetic acid/10% methanol overnight and stained with Coomassie Blue R-350. The images from analytical gels were collected as described previously [13]. Intra-gel spot detection, inter-gel matching and normalization of spot intensities were performed using the Differential In-gel Analysis (DIA) mode and Biological Variation Analysis (BVA) mode of DeCyder 2D software (GE Healthcare, Munich, Germany), respectively. In-Gel Trypsin Digestion of Proteins and Mass Spectroscopic analyses for these experiments were performed as described previously [13].

### Immunoblotting analysis

Each of the proteins identified by 2-D DIGE analyses was confirmed by immunoblot analyses. Protein concentration was determined and immunoblot analyses were performed as described previously [14]. An aliquot of total CSF containing 1-3 μg from each of the subject participants was used for the validation analyses. Mouse monoclonal anti-apolipoprotein-A1, anti- α-1-acid glycoprotein, anti-vitamin D binding protein, anti-cystatin C, and anti-β-hemoglobin and rabbit polyclonal anti-prostaglandin D2 synthase were obtained from Santa Cruz Biotechnology (Santa Cruz, CA, USA). In addition, H-ferritin was quantified using immunoblot analyses. One μg of total protein was used for the analysis and a custom rabbit polyclonal H-ferritin antibody from Covance Biotechnology (Denver, PA, USA) was used as the primary antibody. All immunoblot analyses were run in triplicate.

The immunoblots were quantified using densitometric analysis. Chemiluminescent detection was performed using either anti-mouse or anti-rabbit IgG ECL HRP-conjugated secondary antibodies (GE Healthcare). FujiFilm imaging system was used for detection, and densitometric analysis was performed with Multiguage software version 3.0.

### Statistical analyses

The Extended Data Analysis tool (EDA) in the DeCyder 2D software package was used for the statistical analysis of the 2D-DIGE experiments. Proteins that were significantly altered were identified by Student’s t-test. Protein spots that were differentially expressed (*p* ≤ 0.05, Av. Ratio ≥ 2.0) between RLS/WED and control CSF samples were identified using MALDI-TOF-MS.

Statistical analyses of the validation immunoblot assays were performed using Student’s t-test.

## Results

### CSF protein profile

**Figure 1:**
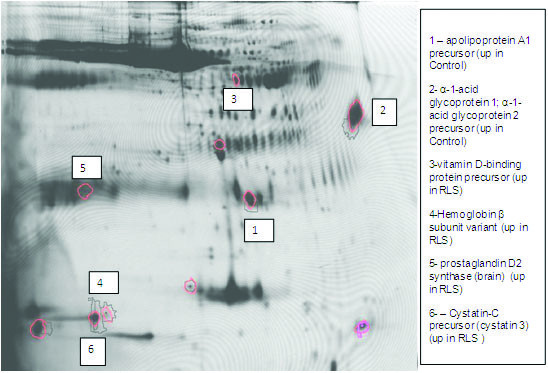
***Representative 2D***-***DIGE analysis of RLS***/***WED versus Control CSF.*** This figure represents a single 2D-DIGE analysis performed on the CSF from RLS/WED and Control subjects. Differentially expressed proteins are encircled in red. Differentially expressed proteins that were able to be identified by MS/MS are listed in the legend.

CSF was obtained from 5 female RLS/WED patients (mean age ±SD 58.0±12.1 years old) and 5 female age-matched controls (mean age ± SD 58.8±7.66 years old) (Table 2). The mean RLS/WED Severity score on the International RLS Study Group (IRLSSG) scale was 31.6±4.15. Analysis of the protein expression using DeCyder-DIA software provided 3-D simulation of the protein spots, allowing an objective view for comparison of spot intensity between the different gels. Reproducibility and consistency in our results was enhanced by performing all analyses at least in duplicate and analyzing the duplicate samples. Approximately 663 protein spots were identified when 20,000 pixels were used as the filter limit in each group of samples. A composite 3-D image is shown for each group in a representative area of the gel (Figure 1). The data from the gels were reviewed by an investigator blinded in regard to the sample identification. Of the 663 spots in the gel that were observed, 6 met the pre-experiment criterion of having at least a two-fold difference in intensity (*p* ≤ 0.05, Av. Ratio ≥ 2.0). These protein spots were chosen for identification by MALDI-TOF. Of the six proteins selected (Table 3), four were increased and two were decreased in the RLS/WED subjects.

**Table 3:** **CSF proteins showing at least a two**-**fold difference in expression between RLS**/**WED and control subjects**

**Identified protein**	**Gi access no.**	**Gene name**	**Function**	**Fold change**	**MW**	**pI**
**Proteins that are down**-**regulated**-**regulated in RLS**/**WED CSF**						
Apolipo protein A-I (precursor)	4557321	APOA1	Major protein component of high density lipoprotein (HDL) in plasma. Promotes cholesterol efflux from tissues to the liver for excretion.	−1.97	30758.93	5.56
Alpha-1-acid glycoprotein 1	112877	AGP	Reduces neutrophil phagocytosis of bacteria, inhibits neutrophil migration and superoxide production, reduces activated platelet aggregation, and inhibits activated lymphocytes proliferation.	−5.25	23724.84	4.93
**Proteins that are up**-**regulated in RLS/WED CSF**						
vitamin D-binding protein (precursor)	181482	GC	Binds vitamin D and its metabolites and transports them to target tissues	2.32	54611.68	5.4
Prostaglandin D2 synthase 21kDa (brain)	55962673	PTGDS	Neuromodulator and trophic factor in the CNS. Involved in smooth muscle contraction and relaxation; and inhibits platelet aggregation.	2.18	23049.57	9.92
cystatin C	181387	CST3	Inhibitor of lysosomal proteinases and an extracellular inhibitor of cysteine proteases	11.32	16047.18	9
β-Hemoglobin	183830	HBB	most common form of hemoglobin in adult humans	4.53	16086.3	6.75

### Immunoblot analyses

**Figure 2:**
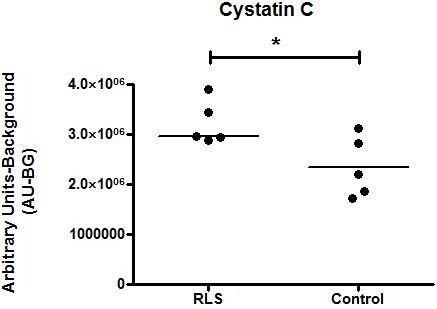
***Immunoblot quantification for cystatin C in CSF of RLS***/***WED and control subjects.*** This figure demonstrates that RLS/WED CSF contains higher Cystatin C levels compared to control subjects. This data is compiled from the CSF from 5 RLS/WED and 5 control patients and each sample was analyzed in triplicate. Single line reflects the median value and the asterisk (*) reflect statistical significance (*p* <0.05) as determined by an unpaired t-test analysis.

Immunoblot validation was performed on all of the candidate proteins. Cystatin C was able to be validated with immunoblot analyses (Figure 2) and was elevated in the RLS/WED group (*p* < 0.05). In addition, because it had been reported that the expression of proteins involved in maintaining iron homeostasis in CSF were altered in CSF in patients with RLS/WED, H-ferritin levels were determined in the CSF using immunoblot analyses but the differences did not reach statistical significance.

## Discussion

Prior to this study, only specific iron-related proteins and dopaminergic metabolites have been examined in the CSF of RLS/WED patients [15,16]. Although the importance of iron in the etiology of early-onset RLS/WED has been established, our proteomic approach in this study permitted identification of proteins that could prove valuable in understanding pathways affected in RLS/WED and identifying targets for treatment strategies.

In this discovery study, we have utilized a 2D-DIGE coupled with tandem MS to identify 6 candidate CSF protein markers for early-onset Restless Legs Syndrome (RLS/WED); four proteins (Cystatin C, Lipocalin-type Prostaglandin D2 Synthase, Vitamin D binding Protein, and β-Hemoglobin) were increased and 2 proteins (Apolipoprotein A1 and α-1-acid Glycoprotein) were decreased in RLS/WED patients compared to controls.

Cystatin C levels were increased in the CSF of early-onset RLS/WED patients. Cystatin C is a cysteine proteinase inhibitor that has multiple functions *in vivo*. It is known to inhibit both endogenous and exogenous cysteine peptidases, regulate the immune system, have antibacterial and/or antiviral effects; and neuroprotective effects during brain injury. Cystatin C has been described previously as a biomarker of pain in lumbar disk herniation, sciatica, and labor [17,18]. The level of cystatin C observed in the CSF of patients with these conditions was found to be dependent on the pain’s intensity and duration. The increased expression of cystatin C in the CSF from RLS/WED patients is consistent with multiple reports of pain associated with RLS/WED symptoms and increased temporal summation to heat pulses in RLS/WED patients suggesting that RLS/WED is associated with amplified CNS pain processing [19,20]. Also, RLS/WED has significant comorbitity with a number of pain conditions such as fibromyalgia [21] and headache [22]. The finding of elevated cystatin C in the CSF of RLS/WED subjects may provide an objective measure of the pain associated with RLS/WED in some of the patients. Also cystatin C has been reported to be elevated in response to inflammation; therefore, there is the potential that immune system activation may be occurring in the CNS of patients with RLS/WED. A recent review by Weinstock *et al*. [23] suggests that some conditions that are associated with RLS/WED have an inflammatory/immune component and that inflammation may be responsible for the CNS iron deficiency.

In this study, we found increased levels of lipocalin-type prostaglandin D2 synthase (L-PGDS) in RLS/WED CSF. L-PGDS is the most abundant protein in the CSF and serves two roles: 1) synthesis of prostaglandin D2 (PGD2) from prostaglandin H2 (PGH2); and 2) binding and transporting small lipophilic molecules. L-PGDS alterations have been reported in a number of sleep disorders including narcolepsy and obstructive sleep apnea with and without daytime sleepiness [24-26] and thus its increase is relevant to sleep disruptions in RLS/WED. We have reported that PGDS is decreased in CSF of monkeys that were iron deficient [13]. Furthermore, the increase in PGDS in RLS/WED CSF is inconsistent with the reports of decreased brain iron status in RLS/WED [14]. We suggest that the increase in PGDS that was observed in RLS/WED patients could be part of a compensatory mechanism. It has been reported that L-PGDS is upregulated in the brains of subjects with hypoxic-ischemic encephalopathy (HIE) [25]. Also, it has been demonstrated in a mouse model of HIE that L-PGDS and HIF-1α expression are co-localized and it was implied that L-PGDS may protect neurons from hypoxic insult by local secretion of PGD2. We could hypothesize, therefore, that the increased L-PGDS in RLS/WED may be a result of hypoxia pathway activation [25]. This hypothesis is further supported by our recent finding of HIF pathway activation in the brains and microvasculature of RLS/WED subjects [27].

In this study, we report elevated vitamin D binding protein in the CSF of RLS/WED subjects. Vitamin D binding protein (VDP) is noted for specifically binding and transporting a number of sterols including vitamin D metabolites to target cells, however, it has been also been shown to be a scavenger of toxins that are released due to cellular injury or death [28] and a modulator of the immune response [29]. Alterations in cerebrospinal fluid levels of VDP have been linked primarily to neurodegenerative diseases, such as multiple sclerosis, Parkinson’s disease, and trauma [17,30,31]. Vitamin D also has important ties to the pathways that have been identified in the etiology of RLS/WED. Vitamin D deficiency has been linked to altered dopamine concentrations in the cortex [32], and blood and tissue iron content has been reported to increase following vitamin D supplementation [33]. Balaban *et al*. have reported decreased serum 25-hydroxyvitamin D levels in female RLS/WED subject and have postulated that the CNS iron deficiency that occurs in RLS/WED may induce alterations in the dopaminergic pathway via a vitamin D-mediated pathway [34]. Because of the tie between iron, dopamine and vitamin D, it is tempting to propose that elevated VDP that we observed in this study could be reflective of the alterations in the iron/dopamine pathway. One could also hypothesize that the elevation of VDP could also be reflective of activation of inflammatory/immune cascades in the CNS of RLS/WED subjects as further supported by the alterations of cystatin C and alpha-1 acid glycoprotein levels in RLS/WED CSF.

Beta-Hemoglobin was found to be increased in RLS/WED CSF. β-Hemoglobin is a major component of adult hemoglobin and normally is not present in the CSF. As such, the presence of β-Hemoglobin in the CSF is often considered to be indicative of blood contamination. While, obtaining a CSF tap with no blood contamination is extremely difficult, we have examined the CSF for frank signs of blood to rule out contamination. β-Hemoglobin is known to transport oxygen in the blood, however, it has also been reported to be important in dopamine regulation in the brain [35]. Biagioli and colleagues have reported that overexpression of Hbb-b1 and Hba-a1 (the genes encoding Hemoglobin β and Hemoglobin α) in dopaminergic cell lines alters transcription of genes related to iron metabolism, oxygen homeostasis, oxidative phosphorylation, and nitric oxide synthesis [35]. Because evidence exists for the disruption of both the dopaminergic and oxygen homeostatic systems in RLS [27,36-38], it is tempting to suggest the finding of increased β-hemoglobin in the RLS/WED CSF may reflect a more chronic condition in RLS/WED and perhaps may point to a mechanistic explanation for the altered iron, dopamine and oxygen protein profiles that are observed in RLS/WED.

Apolipoprotein A1 was found to be decreased in the CSF of patients with RLS/WED. Apolipoprotein A1 is the primary protein constituent of high density lipoproteins (HDL). Although the role of Apolipoprotein A1 in the CNS is not completely understood, it is thought to be important for transport of lipids into the brain [39]. To date, no association has been reported between Apo-A1 and RLS/WED, however, recent studies [40,41] have described a higher prevalence of hypercholesterolemia in RLS/WED subjects. Decreased serum Apo-A1 levels have been linked to increased incidence of cardiovascular disease [42] which, if the decrease in Apo-A1 that was observed in RLS/WED CSF was reflective of a global Apo-A1 level, this could be related to the increased risk for cardiovascular disease in RLS/WED [4-7].

Alpha-1-acid glycoprotein levels were decreased in the CSF from RLS/WED patients. Alpha-1-acid glycoprotein (AGP) is synthesized primarily in the liver, but also is reportedly synthesized in the endothelial cells of the blood–brain-barrier. Alpha-1-acid glycoprotein (AGP) is an acute phase reactant that is involved in the inflammation cascade and is thought to provide immediate and effective response to inflammatory damage prior to successive protective mechanisms [43]. Their protective functions seem to result mainly from iron sequestration [44,45]. Our finding of decreased AGP in the CSF of RLS/WED subjects is reflective of decreased iron content in the CNS and provides further support for altered iron homeostasis in the brains of RLS/WED patients [14,46,47].

## Conclusion

Our results reveal a protein profile in the RLS/WED CSF that is consistent with iron deficiency, dopamine dysregulation and inflammation. The APO-A1 finding may be of relevance outside of CNS given the recently reported increased risk of cardiovascular disease in patients with RLS and cardiovascular dysfunction and reports of painful symptoms. The profiles in the CSF are also consistent with neuropathological findings of activation of HIF pathways and alterations in dopaminergic systems. The data indicate that the CSF protein profile, if confirmed in larger sample sizes, may provide support for existing hypotheses about a biological basis for RLS/WED which could prove clinically meaningful in evaluating therapeutic strategies and identifying new targets.

## Competing interests

Dr. Allen has in the last 2 years served as a consultant for Boehringer Ingelheim, GlaxoSmithKline, Luitpold Pharmaceuticals, Pfizer, EMD Serono, Pharmacosmos, Neurogen, Jazz Pharmaceuticals and UCB Pharma. He also received research support from GlaxoSmithKline, Pharmacosmos and the USA National Institutes of Health. Dr. Earley was a member of the Data Safety Committee for Phase III clinical trial by Merck. Dr. Connor discloses that he is a paid consultant for GlaxoSmithKline and the International Copper Association and has consulted for Neurogen. Drs. Patton and Cho, and Mr. Clardy report no disclosures.

## Authors’ contributions

SMP carried out the 2D-DIGE analyses, identification of biomarker proteins, and drafted the manuscript, YWC recruited, diagnosed and obtained cerebrospinal fluid samples from RLS/WED and control subjects, TC performed the DeCyder-DIA proteein analyses of 2D-DIGE images, RPA participated in study design and coordination, and assisted in the draft of the manuscript, CE participated in study design and assisted in the draft of the manuscript, and JRC participated in the study design and assisted in the draft of the manuscript. All authors have read and approved the final version of the manuscript.
